# Causes and Consequences of HPV Integration in Head and Neck Squamous Cell Carcinomas: State of the Art

**DOI:** 10.3390/cancers13164089

**Published:** 2021-08-13

**Authors:** Harini Balaji, Imke Demers, Nora Wuerdemann, Julia Schrijnder, Bernd Kremer, Jens Peter Klussmann, Christian Ulrich Huebbers, Ernst-Jan Maria Speel

**Affiliations:** 1 Department of Otorhinolaryngology, Head and Neck Surgery, Faculty of Medicine, University of Cologne, Kerpener Strasse 62, 50931 Cologne, Germany; nora.wuerdemann@uk-koeln.de (N.W.); jens.klussmann@uk-koeln.de (J.P.K.); christian.huebbers@uk-koeln.de (C.U.H.); 2 Jean-Uhrmacher-Institute for Otorhinolaryngological Research, University of Cologne, Geibelstrasse 29–31, 50931 Cologne, Germany; 3 Department of Pathology, GROW-School for Oncology and Developmental Biology, Maastricht University Medical Center, Universiteitssingel 40, 6229 ER Maastricht, The Netherlands; imke.demers@mumc.nl (I.D.); j.schrijnder@student.maastrichtuniversity.nl (J.S.); ernstjan.speel@mumc.nl (E.-J.M.S.); 4 Center for Molecular Medicine Cologne (CMMC), Faculty of Medicine, University of Cologne and University Hospital Cologne, Robert-Koch-Strasse 21, 50931 Cologne, Germany; 5 Department of Otorhinolaryngology Head and Neck Surgery, GROW—School for Oncology and Developmental Biology, Maastricht University Medical Centre, P. Debyelaan 25, 6229 HX Maastricht, The Netherlands; bernd.kremer@mumc.nl

**Keywords:** high-risk human papillomaviruses, head and neck squamous cell carcinomas, viral DNA integration, PCR, DIPS-PCR, APOT-PCR, WGS, WES, capture-based assay, RNASeq, FISH, consequences of HPV integration

## Abstract

**Simple Summary:**

In human papillomavirus (HPV) associated head and neck squamous cell carcinomas (HNSCC) s, the HPV genome is commonly found integrated in the human genome. The event of viral–human genome integration may act as a driver of carcinogenesis. Hence, it is vital to assess the viral integration status of a tumor. In this review, current and emerging techniques for integration detection are thoroughly discussed with their advantages and disadvantages. Additionally, the review also discusses the causes of HPV integration into the cellular genome, as well as its ramifications, impacting possible clinical implications.

**Abstract:**

A constantly increasing incidence in high-risk Human Papillomaviruses (HPV)s driven head and neck squamous cell carcinomas (HNSCC)s, especially of oropharyngeal origin, is being observed. During persistent infections, viral DNA integration into the host genome may occur. Studies are examining if the physical status of the virus (episomal vs. integration) affects carcinogenesis and eventually has further-reaching consequences on disease progression and outcome. Here, we review the literature of the most recent five years focusing on the impact of HPV integration in HNSCCs, covering aspects of detection techniques used (from PCR up to NGS approaches), integration loci identified, and associations with genomic and clinical data. The consequences of HPV integration in the human genome, including the methylation status and deregulation of genes involved in cell signaling pathways, immune evasion, and response to therapy, are also summarized.

## 1. Introduction

Head and neck squamous cell carcinoma (HNSCC) is presently the sixth leading type of cancer worldwide, with 630,000 new patients resulting in over 350,000 deaths annually [1]. Generally, HNSCC originates from the mucosal linings of the upper aerodigestive tract. In more than 90% of the cases, HNSCCs arise in the oral cavity, oropharynx, and larynx [1,2], frequently due to the activation of oncogenes such as epidermal growth factor receptor (*EGFR*), as well as loss-of-function mutations in tumor-suppressor genes such as *TP53* and *CDKN2A* [3]. Treatment of early-stage HNSCC usually comprises surgery and/or radiotherapy. However, for patients with advanced HNSCC, multimodal treatment regimens such as surgery followed by radiation or definitive platinum-based chemoradiation are performed [2,3]. Additionally, in advanced and/or metastasized HNSCC, targeted therapy with the EGFR specific monoclonal antibody Cetuximab or immunotherapy using anti-PDL1 antibodies may be incorporated into the patient treatment regime [2,4,5,6]. Patient treatments unfortunately cause early and late toxicity which severely lower the quality of life [4]. Moreover, preneoplastic sites often persist after treatment, allowing the possibility of local recurrences and second primary tumors which are both responsible for a large proportion of deaths [2].

HNSCC carcinogenesis can be majorly classified into HNSCC mediated by high-risk human papilloma virus (HPV) infection and HPV-negative HNSCC that is primarily caused by tobacco and alcohol consumption [7]. Over the last decade, a striking increase in HPV-positive HNSCC incidences has been observed in the Western world [2], especially of oropharyngeal squamous cell carcinoma (OPSCC)s. Up to 90% of the OPSCCs have been associated with HPV [8]. Furthermore, it has been reported that, in the USA, the incidence of HPV-positive HNSCCs has surpassed that of HPV-positive cervical SCCs [9,10].

Despite the morphological (e.g., poorly differentiated), molecular (e.g., less chromosomal aberrations), and clinical characteristics (e.g., younger age, less tobacco and alcohol consumption) of HPV-positive tumors, patients with this type of HNSCC have a favorable prognosis, regardless of the treatment strategy applied [2,4,11]. This could be attributed to the fact that HPV-positive patients present with fewer genetic alterations, an impaired DNA double strand break repair response, and respond better to radiotherapy due to an intact apoptotic response [11]. The above are likely to be caused by single tumor-initiating events rather than field carcinogenesis. This is generally observed with younger and healthier age groups and hence they display fewer comorbidities. Moreover, radiotherapy and chemotherapy could trigger an immunological response against virus-specific antigens [12]. Nevertheless, additional risk factors such as smoking, EGFR overexpression, advanced nodal stage, and chromosomal instability can cause poor prognosis in patients with HPV-positive HNSCCs [8].

For a biologically relevant HPV infection, a couple of events are considered to be essential. Sites of infection involve stratified keratinocyte layers of epidermal origin. The virus particularly prefers functional epithelial appendages, such as salivary glands in the oral cavity and tonsillar crypts, as well as sites where stratified epithelium is adjacent to columnar epithelium, for instance in the uterine cervical transformation zone [13]. These sites are thought to be preferentially targeted because they lack the highly structured barrier function of the epithelium and have an increased occurrence of epithelial reserve cells/stem cells. To hijack these cells, wounds/microlesions are furthermore required to reach the basal cell layer so that it is ensured that actively proliferating cells become infected. At the sites of (micro)injury, an influx of serum containing Heparan sulfate proteoglycan (HSPGs), growth factors (GFs), and cytokines are produced to promote wound healing. Subsequently, HPV L1 capsid protein binds to exposed HSPGs [14]. In addition, virions binding to α6-integrins is required, initiating further intracellular signaling events. In turn, conformational changes induced in HSPGs result in L2 cleavage, binding of the exposed L2 N-terminus to an L2-specific receptor (annexin A2 heterotetramer), and subsequent clathrin-, caveolin-, lipid raft-, flotillin-, cholesterol-, and dynamin-independent endocytosis of HPV16 [15].

**Figure 1:**
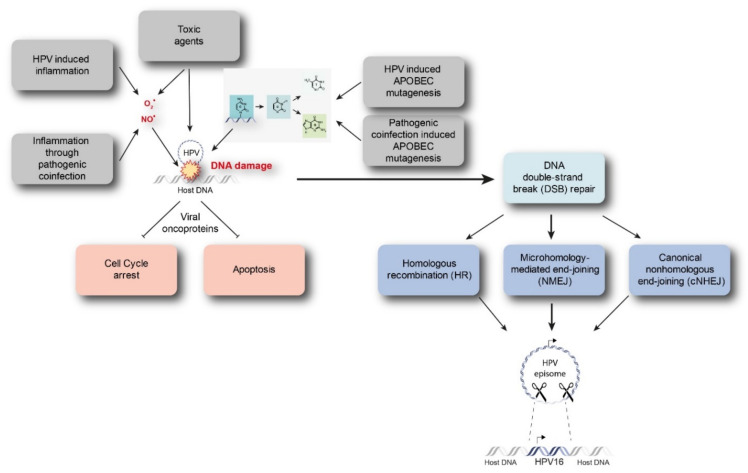
Discussed drivers of DNA damage and HPV integration. Intrinsic and extrinsic drivers such as inflammation, toxic agents, or APOBEC mutagenesis caused by HPV infection are able to instigate DNA damage. Subsequently, chromosomal aberrations and DNA damage repair mechanisms might promote viral integration. APOBEC = Apolipoprotein B mRNA-editing catalytic polypeptide.

**Figure 2:**
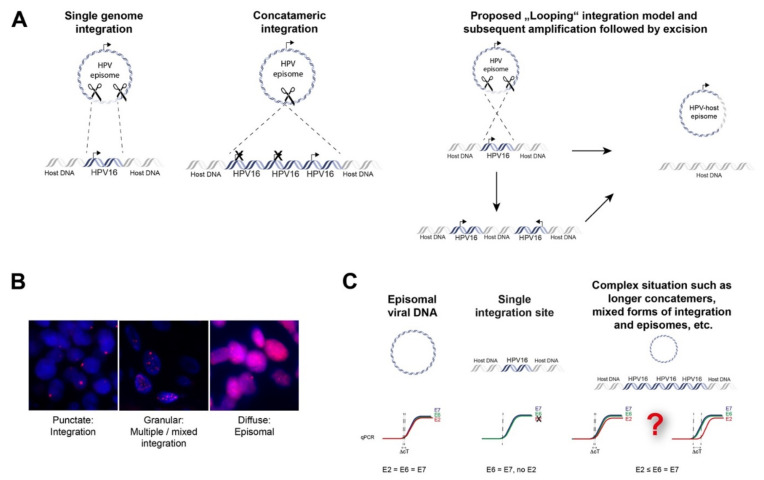
Mechanisms of episomal HPV-DNA integration into the human genome and non-sequencing based methods to prove integration. (**A**) Direct integration of a single viral genome into the host genome; direct integration of concatamerized viral genomes and proposed “Looping” integration of the viral genome with recurrent patterns of focal amplification and rearrangements next to the integration sites which finally may lead to excision and loss of viral DNA or viral-human fusion episomes; (**B**) fluorescence in situ hybridization with probes against HPV16 of tumor cells depicting integrated, mixed episomal and integrated and episomal status, magnification 100×; (**C**) qPCR strategy to analyze viral integration. An E2/E6 copy number ratio ≠ 1 may indicate disrupted E2 and viral integration. However, concatamerized HPV-genomes and/or additional HPV-episomes with several full-length E2 copies together with a single disrupted E2 gene will be challenging to detect.

Starting from a transient HPV infection, the viral genome maintains as extra-chromosomal episomes. However, persistent infection by high-risk HPVs may lead to the integration of viral genome into the host genome. Viral integration requires both viral and host DNA breakage. Therefore, the rate of integration is expected to be related to the degree of DNA damage, which can be induced by a number of factors (Figure 1) [15,16]. In particular, excessive amounts of reactive nitrogen and oxygen species originating, for example, from inflammation caused by HPV infection itself (especially through the expression of E6 and E7) or from coinfection with other pathogens, as well as toxic agents originating from environmental or other sources, can cause DNA damage [17,18,19]. In addition, Apolipoprotein B mRNA-editing catalytic (APOBEC) polypeptides are recently identified as a source of DNA damage, as will be discussed later. Subsequently, there is accumulation of chromosomal alterations and activation of DNA damage repair mechanisms that could promote viral integration. Two possible mechanisms have been proposed by which integration occurs, namely direct insertion and looping integration (Figure 2).

Direct insertion is thought to occur by a process known as microhomology-mediated end-joining (MMEJ), which can be caused by the interference of HPV oncoproteins with the DNA double-strand break (DSB) repair pathway. MMEJ is highly error-prone and acts as a backup pathway for defects that occur in the homologous recombination (HR) pathways or major canonical non-homologous end-joining (cNHEJ) [20]. This can lead to repair events that are lethal. Interestingly, increased microhomology has been observed between HPV virus and viral integration genomic sites in oropharyngeal and cervical cancers, signifying a role of MMEJ. This is achieved when the broken viral genome exploits sequence homology, i.e., identical genomic nucleotide sequence, between the viral ends and the host genome. This is followed by deletion of these microhomologies from both genomes and insertion of the viral genome as a single genome or as concatemerized genomes into the host genome [21]. The DNA looping integration model proposes recurrent patterns of focal amplification and rearrangements, resulting in concatemers present downstream from the integration sites. This suggests that concatemers of the host and viral genomes become amplified in tandem and are reinserted back into the host genome [22]. Moreover, this may explain extrachromosomal virus-host fusion episomes that can arise when looping integration occurs without reinsertion [21].

Integration of the viral genome into the host genome often leads to deletion or truncation of the viral gene E2, resulting in loss of E2 transcript production. This in turn facilitates deregulated transcription of the viral E6 and E7 oncogenes, leading to ubiquitous expression of the corresponding E6 and E7 proteins [21]. Subsequently, this leads to deregulation of many cellular processes, including cell proliferation and apoptosis, for example by inactivation of the tumor-suppressors p53 and pRB [1,2,3,4,7,8,9,10]. Despite this knowledge, it is still unclear whether the integration of HPV into the human genome is associated with distinct biological consequences. Moreover, the association between HPV integration and poor patient outcomes is still debated, and results are controversial. Furthermore, tumors with a mixed viral physical status have been identified, posing the question whether or not these tumors show different biological behavior than tumors with solely integrated or episomal virus. This work aims to summarize the recent literature and adds to the knowledge of three reviews on HPV integration in HNSCC [15,21,23].

## 2. Materials and Methods

To find relevant literature on the causes and consequences of HPV integration in HNSCC, a detailed search was performed in the PubMed database (https://pubmed.ncbi.nlm.nih.gov, accessed 5 July 2021) using the search terms indicated in Appendix A. The timeframe of this analysis was fixed, by including papers published between January 2016 and April 2021. This systematic search resulted in a total of 101 papers, which were evaluated by reading the abstract followed by the full text (H.B. and I.D.). Thirty-six papers were eventually included in this study because they contained information about the physical status of HPV (episomal, integration) and HNSCC. One paper was included by screening references of the selected papers. To provide information, advantages and disadvantages of techniques to detect viral integration, 11 additional papers were included from PubMed database using search terms describing the different techniques

## 3. Results

### 3.1. Involvement of APOBEC Mediated Anti-Viral Defense in HPV Integration

Besides known mechanisms that can lead to DNA damage as represented in Figure 1, recent literature has provided evidence that Apolipoprotein B mRNA-editing catalytic (APOBEC) polypeptides are likely involved in HPV integration. APOBECs represent a family of 11 DNA cytosine deaminases that are a vital arm of the innate immune response. They potently inhibit retrovirus, transposon, and DNA virus replication. APOBECs catalyze the deamination of cytidine in both DNA and RNA. Inappropriate APOBEC expression has been identified as a genomic mutator that can eventually cause cancer [24]. Kondo et al., have reported that APOBECA3A (A3A) or A3B (A3B) expressions are involved in replication inhibition and increases the number of double strand breaks [24]. This in turn induces genomic instability and causes favorable circumstances for viral integration. Moreover, they found that A3A can catalyze the hypermutation of viral E2 and further state that A3A-induced deamination may increase the chance of viral integration Furthermore, supporting the results of Kondo et al., it was observed that the expression of A3B was found to be significantly higher in HPV-positive HNSCCs than in HPV-negative HNSCCs [25]. This additionally suggests that the high A3B expression in HPV-positive HNSCCs can cause beneficial genomic conditions allowing HPV integration. In conclusion, this association between APOBEC induced mutational signatures and HPV suggests that an impaired antiviral defense is a driving force in HPV-positive HNSCCs [25].

### 3.2. Approaches to Detect HPV Integration in Tumor Tissue

**Figure 3:**
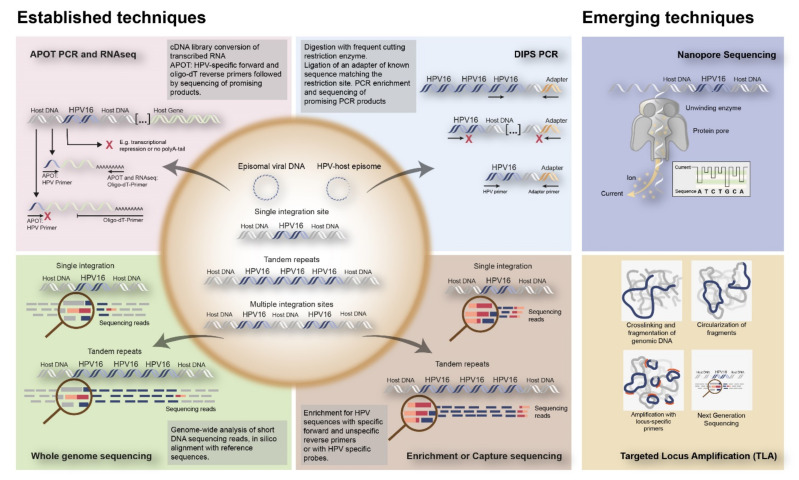
Overview of established and emerging techniques to detect HPV integration into the human genome. The established techniques to detect integration include RNA based techniques such as APOT PCR and RNAseq; DNA based techniques such as DIPS PCR, WGS, and Enrichment or Capture sequencing. Nanopore Sequencing and TLA are represented as emerging techniques for HPV integration detection. APOT = amplification of papilloma virus oncogene transcripts assay; DIPS-PCR = Detection of integrated papillomavirus sequences by ligation-mediated PCR; RNAseq = RNA sequencing.

To date, several techniques have been used to detect HPV integration in tumor tissue. Initially, approaches included in situ hybridization (ISH) or fluorescence in situ hybridization (FISH), which could visualize HPV DNA or RNA as well as viral integration at the single cell level in cells and tissues (Figure 2B). Alternatively, PCR-based techniques have been developed, including quantitative PCR (qPCR), which determines E6/E7 copy numbers in relation to E2, Detection of Integrated Papillomavirus Sequences (DIPS) PCR which detects virus-human DNA sequences, and Amplification of Papillomavirus Oncogene Transcripts (APOT) PCR, which detects virus–human RNA transcripts (Figure 2C and Figure 3).

In addition, Next Generation Sequencing (NGS) techniques have been coming of age, including Whole Genome Sequencing (WGS), Whole Exome Sequencing (WES), and RNASeq, all identifying HPV-human nucleic acid sequences (Figure 3).

Emerging techniques are being developed, investigating viral integration in combination with HPV sequences capturing utilizing HPV-specific custom-made RNA probes. This enables DNA enrichment for viral sequences, increasing the chance to find HPV integration. This enrichment step is followed by amplification and NGS [17,18,19]. Examples of emerging techniques to detect HPV integration are nanopore sequencing on DNA/RNA isolated from fresh frozen tissues, combining HPV capturing with long read sequencing, as well as Targeted Locus Amplification (TLA) on DNA isolated from FFPE tissues, combining HPV capturing with circularization of DNA fragments and amplification (Figure 3). An overview of all the currently used techniques to identify HPV integration, as well as their advantages and disadvantages, are given in Table 1.

As mentioned above, an increasing number of studies have employed NGS techniques to determine the presence and location of the HPV integration in the human host genome. Inherent to reliable NGS data is an optimal bioinformatic pipeline that ensures rapid and exclusive detection of the viral genome from the large-scale genome-wide DNA sequencing of the cancer genome, typically by detecting virus-host chimeric fusions or paired-end reads [21]. Various bioinformatical approaches to identify viral integration sites have been described in the literature, including VirusSeq, VirusFinder, SurVirus, VirTect, HIVID2, and HGT-ID, which have been used to detect integrated HPV genomes specifically [39,40,41,42,43,44,45,46,47,48,49]. The variety of viral integration detection software tools might at least partly explain the broad range in the number of reported HPV integration sites (0–600) in cervical cancers [22,50,51]. It has been suggested that these high integration rates are a result of a low-stringency bioinformatics approach [21]. When mapping integration sites, multiple aspects that may induce artifacts in bioinformatic data should be considered. For example, splicing from within the HPV genome into the distal host genome could result in a fusion transcript, which can be misidentified as a breakpoint. In addition, sequencing machine contamination could lead to overestimation of HPV integration sites and bioinformatic tools may not be able to differentiate between reads from circularized (episomal) sequences and linearized genome sequences. Furthermore, artifacts could be introduced due to microhomology sites, duplicate reads, mitochondrial genomes integrating in a highly similar manner as human genomic DNA, and mismatch bases. Hence, there is a necessity for quality control of the bioinformatics data and confirmation of integration sites by other established techniques [21,52].

As a consequence, newly developed bioinformatic tools have recently been described in the literature, of which some examples will be explained below. Viral integration and Fusion identification (ViFi) has been presented as a new tool in detecting viral integrations from WGS data and human–virus fusion mRNA from RNAseq data. Unlike other bioinformatic pipelines that only use reference-based alignment mapping to identify viral reads, ViFi combines this with a phylogenetic model of HPV families to better detect evolutionarily divergent viruses [53]. An approach that detects Virus integration sites through Reference Sequence customization (VERSE) was first described in 2015 and is designed to ‘correct’ human reference genomes to create a new ‘personalized’ human reference genome, which aims to improve alignment of short reads and thereby virus detection sensitivity through WGS, RNAseq, and targeted sequencing [25,54]. A number of capture-based sequencing methods have been reported with bioinformatics tools. For example, nanopore sequencing distinguishes itself from other sequencing techniques as it enables sequencing of extremely long DNA molecules. This is at the cost of less sequence accuracy and the inability to sequence relatively short DNA and RNA isolated from FFPE material (Table 1). Specifically designed bioinformatic methods are being developed to analyze the entire ultra-long sequencing reads and to perform error correction of the sequence data [36]. Furthermore, a novel pipeline, specifically for targeted capture sequencing data, has been generated, referred to as SearcHPV [55]. It has shown to operate in a more accurate and efficient manner than existing pipelines on capture sequencing data, something which has been lacking in the field. Another advantage of this software is that it performs local assembly of overlapping DNA segments around the junction site, which simplifies confirmation experiments.

Cameron et al. developed a virus-centric approach, called VIRUSBreakend. This tool uses single breakends, breakpoints in which only one side can be unambiguously placed to the reference genome, with the advantage that viral integration can be detected in regions of low mappability, such as centromeres and telomeres. VIRUSBreakend first identifies the viral genome within the host genome, compares this to viral NCBI taxonomy IDs, selects a viral reference genome based on sequence similarity, and aligns all read pairs with this viral reference genome. Subsequently, single breakends are assembled and host integration sites are identified [56].

### 3.3. Prevalence of HPV Integration

Uterine cervical SCCs are HPV-positive in 95–100% of the cases with varying frequencies of integration for different HPV subtypes. HPV16 tends to integrate in 50–80% of the cases and HPV18 in >90% [15,21,32]. In OPSCCs, HPV positivity ranges from 20–90% in different studies depending on geographical location, sample preparation, and detection method used, and, furthermore, 90–95% of virus-positive OPSCCs are infected with HPV16 [44,57].

Using FISH with whole virus genome probes, HPV integration percentages of 40–60% were described for OPSCCs [58]. An integration incidence of 40–100% was reported in tonsillar squamous cell carcinomas (TSCC)s using DIPS and APOT PCR techniques [32,59]. Recent literature describing E2, E6/E7 qPCR based HPV integration detection shows lower integration percentages (5–25%), dependent on anatomical tumor location, and a larger proportion of tumors containing both integrated and episomal HPV DNA (40–85%) [37,50,51,52,58,60,61]. Integration rates determined with NGS-based techniques range from 15% to 70% [60,62]. However, the number of included patients is often low and the majority of studies included tumors originating from multiple locations, also outside the oropharynx. In addition, often no distinction is made between solely integrated HPV and the mixed form, in which episomal DNA is also present. These aspects, among others, make it difficult to directly compare studies and observed integration rates. Furthermore, differences in applied bioinformatic pipelines to detect viral integration might also contribute to divergent integration rates, as mentioned before.

### 3.4. Low HPV Copy Numbers Are Associated with Integration in Liquid Biopsy

Recent research has shown that HPV DNA can also be efficiently detected in liquid biopsies (blood plasma, saliva), as part of the cell free DNA (cfDNA) fraction, and it is a promising biomarker for detection of early primary OPSCCs especially in groups of high risk patients [63]. cfDNA comprise DNA fragments of 160–180 base pairs, released in the blood by processes including apoptosis, necrosis, and secretion. Up to 0.1–1% of this cfDNA may consist of circulating tumor DNA. Plasma circulating tumor HPV-DNA (ctHPVDNA) can be measured over time to analyze the response of the tumor during cancer therapy using multianalyte digital PCR assays. Chera et al. investigated whether ctHPVDNA levels were associated with tumor HPV copy number and HPV physical state using digital droplet PCR [64]. In this study, the prevalence of HPV was observed in 44 patients from a total of 103 patients with OPSCC. HPV status was unknown in 49 patients though all tumors were p16^INK4A^ positive. Their results show that low baseline levels of ctHPVDNA (≤200 copies/mL) were significantly associated with lower tumor HPV copy number (*p* = 0.04). In addition, low tumor HPV copy number (≤5 copies/haploid genome) was significantly associated with HPV integration (*p* = 0.02). From this, it can be concluded that low base-line levels of ctHPVDNA are indicative for low tumor HPV copy number and a greater probability of HPV integration. However, in this study, only 8 out of 20 HPV16-positive patients showed viral integration. Further studies are required to investigate this correlation in a larger sample size and/or the possibility to detect HPV-human DNA fusions in plasma derived cfDNA by NGS.

Similarly, Tang et al. investigated whether HPV integration could be detected in saliva of OPSCC patients using qPCR analysis. They found a significant association between salivary HPV16 load (>10 copies/50 ng) and advanced disease stages [59]. Moreover, they identified mixed or fully integrated HPV in the saliva of 4 out of 127 OPSCC patients of which 74 patients harbored HPV16 DNA and 89 patients showed p16^INK4A^ staining. Even though this number is small and no correlation with disease stage was observed, the authors suggest that these results should be analyzed in a larger cohort.

### 3.5. Loci of HPV Integration in the Human Genome

Molecular studies have provided evidence that ≥1 integration site (s) can be detected in HPV-positive cancers, including HNSCC [15,65]. HPV integration sites are distributed all over the human genome and often lie within or close to fragile sites. HPV integration hotspots have been found in chromosome 2q22.3, 3p14.2, 3q28, 8q24.22, 9q22, 13q22.1, 14q24.1, 17p11.1, and 17q23.1–17q23.2 [65,66]. Interestingly, Walline et al., investigated if integration sites differed for oropharyngeal tumors comparing 10 HPV16 positive patients including five patients who responded well to therapy and five patients whose tumor persisted and recurred [67]. They found that, in responsive tumors, HPV often integrates in intergenic regions, whereas recurrent tumors exhibited complex HPV integration patterns in cancer-associated genes. HPV integration is most frequently detected in genic regions, most often cancer-related genes, such as oncogenes (e.g., *TP63*, *MYC*, *ERBB2*) or tumor suppressor genes (e.g., *BCL2*, *FANCC*, *HDAC2*, *RAD51B*, *CSMD1*) and to a lesser extent in miRNA regions [21,23]. For example, Parfenov et al. studied 279 HNSCC samples in which 35 patients were high risk HPV positive. They observed HPV integration in a known gene among 54% of HPV-positive OPSCC, and 17% within 20 kb of a gene [60]. Similarly, Olthof et al. analyzed 75 HPV16 OPSCC samples and identified 37 integration sites in 29 OPSCC, of which 27 were in known or predicted genes, including 17 with a known role in tumorigenesis [32]. Based on these data, amongst others, it is suggested that HPV integration is not simply a random event, but rather prefers less protected and more accessible chromosomal regions, including highly transcribed (cancer) genes [15].

An interesting finding using HPV integration detection for studying the clonal relationship between bilaterally developing TSCCs was reported by Pinatti et al. [68]. In a case study, six integration events were detected by DIPS-PCR, including two intragenic events in the genes *CD36*, involved in fatty acid import and *LAMA3*, involved in cell adhesion, migration and differentiation of keratinocytes. No identical integration sites were observed between the left and right TSCC. However, it is remarkable that both TSCCs contained HPV16 integration in *CD36*, although slightly different with respect to the genomic location, i.e., intron 5 vs. intron 6. Although the authors suggested this finding as one of the events pointing to a clonal relation between both TSCCs, further mutational profiling of cellular genes and transcripts and access to samples other than FFPE tissue with better quality DNA/RNA are required to provide more evidence for the clonal nature of both TSCCs.

### 3.6. Consequences of Viral Integration

#### 3.6.1. Deregulated Viral Gene Expression

Based particularly on cell transfection studies, the general view is that, upon viral integration, the viral episome is most frequently opened in the E2 open reading frame. This often leads to deletion of E4 and E5 and part of E2 and L2 [13,14,66]. Deletion of E2 disrupts its transcriptional repressor function in the viral Long Control Region (LCR), leading to upregulation of E6 and E7 and subsequent deregulation of cell signaling pathways, increased cellular proliferation and inhibition of apoptosis [11,21]. Interestingly, Reuschenbach et al. found from a total of 57 patients with HPV-positive OPSCC that 16 samples with undisrupted E2 are associated with methylation of E2 binding sites (E2BS3 and E2BSx4) in the LCR, leading to loss of protein expression, pointing to the same effect as deletion of the E2 gene. In most of the latter cases, the LCR was not methylated [69].

More recent studies reported that viral genome methylation is not per se associated with HPV physical status. Although hypermethylation within the LCR was reported in two cell lines (UM-SCC-47 and CaSki), two other cell lines (UM-SCC-104 and SiHa) with a mixed physical status of the HPV genome contained a unmethylated LCR [70]. In this respect, Hatano et al. observed that the methylation status of the integrated HPV genome in three HNSCC cell lines (UPCI:SCC090, UPCI:SCC152, and UPCI:SCC154) correlated to the methylation status of the host genome flanking the integration breakpoints [71]. As a consequence, they suggested that viral (onco)gene expression might be dependent on the location of integration.

Nevertheless, multiple studies on primary tumors have shown that disruption of E2 upon viral integration will not per se lead to increased expression of E6 and E7 oncogenes, suggesting that constitutive rather than high-level expression of viral oncogene transcripts is required in HPV induced carcinogenesis. In tumors with episomal HPV, constitutive expression of E6 and E7 has also been reported [2,58,72,73,74,75].

#### 3.6.2. Deregulated Human Gene Expression

**Figure 4:**
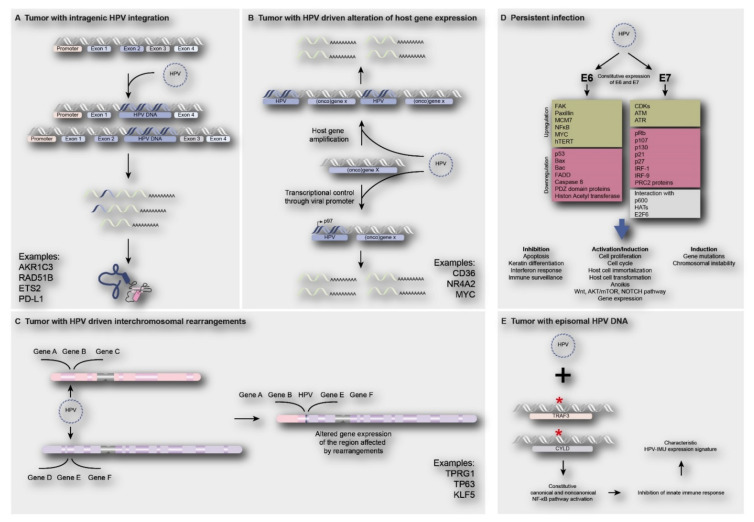
Direct and indirect consequences of HPV infection on human gene expression. (**A**) Integration of HPV in intragenic regions of the human genome causing loss of gene function and/or truncated proteins e.g., *AKR1C3*, *RAD51B*, *ETS2* and *PD-L1* [22,32]; (**B**) integration of HPV near proto-oncogenes such as *CD36*, *NR4A2* and *MYC*, leading to oncogene activation, such as gene amplification or upregulation of gene expression [23]; (**C**) HPV integration may lead to interchromosomal rearrangements, amplification of genes and subsequent increase in expression of genes such as *TP63*, *TPRG1* and *KLF5* [22,32,60,76]; (**D**) The constitutive expression of E6 and E7 oncoproteins upon HPV infection (independent of physical status) will lead to deregulation of cell signaling pathways, inhibition of apoptosis, activation of cell proliferation and induction of gene mutations or chromosomal instability [11,77]; (**E**) Tumors harboring episomal HPV often show the presence of *TRAF3*/*CYLD* mutations leading to constitutive activation of NF-ϰB, resulting in inhibition of innate immune responses, which is a characteristic of HPV-immune response and mesenchymal cell differentiation (HPV-IMU) signature types [78].

Besides the effects on viral oncogene expression, HPV integration might also directly or indirectly affect the host genome. Direct involvement of viral integration on human gene expression may occur when the virus is integrating in or adjacent to a cancer gene, thereby (in) activating its expression. Integration in a tumor suppressor gene might result in loss of gene function, with loss of the wildtype gene on the other chromosome, or translation of truncated proteins. Integration adjacent to an oncogene could lead to gene amplification or enhanced expression from the viral promotor. Additionally, intra -or interchromosomal rearrangements followed by altered expression of genes in these regions might occur. Figure 4A–C shows a number of examples of reported genes directly affected by viral integrants [8,15,22,23,60]. Alternatively, human gene expression may be indirectly deregulated by ubiquitous E6 and E7 expression, independent of HPV physical status. Figure 4D shows reported examples and consequences of indirect deregulation of cellular pathways and processes by HPV infection. Below, examples from the recent literature are described.

#### 3.6.3. Deregulated Expression of the Targeted Gene by HPV Integration

Hassounah et al. showed that HPV is able to integrate into the *CD274* gene encoding Programmed Death Ligand 1 (PD-L1), specifically in front of the sequence coding for the transmembrane domain of the protein (within the intron after exon 4) [79]. This results in transcription of a truncated isoform of PD-L1 that is unable to bind to the membrane but is rather secreted by the cell, as confirmed in vitro using cell lines and transfection experiments. The truncated isoform of PD-L1 maintains its ability to bind to PD-1, inducing a negative regulation of T cell function outside of the cell, which was confirmed by inhibition of IL-2 and IFN-γ secretion.

Additionally, Koneva et al., also identified three tumors in which *CD274* was used as an HPV integration site (integrations within intron 4 and two ‘enhancer sites’ upstream of *CD274)*, which correlated with upregulated PD-L1 expression [80].

Broutian et al. observed HPV insertions flanking a 16-fold somatic amplification of the gene *PIM1* (Proviral insertion site for Moloney murine leukemia virus MuLV) in the HNSCC cell line UPCI:SCC090, in which more integration sites have been identified [8,22]. This amplification was accompanied by an increase of PIM1 transcripts [81]. PIM1 overexpression has been identified in HNSCCs and has been associated with poor survival [82,83,84]. PIM kinases are involved in cellular transformation and substrates of PIM kinase phosphorylation are involved in cell cycle progression, cell growth, and cell death. PIM1 activation causes phosphorylation of several substrates of the PIK3CA/AKT/mTOR pathway, which in turn promotes an increased activation of this pathway and allows increased cell metabolism and growth [81].

A case report published by Huebbers et al. describes a very rare malignant transformation of juvenile-onset recurrent respiratory papillomatosis of the larynx [85]. They reported that the tumor contained integration of low-risk HPV type 6 in the Aldo-Keto Reductase 1C3 (*AKR1C3*) gene, deletion of the corresponding chromosomal region 10p14–10p15.2, and loss of AKR1C3 protein expression [76].

#### 3.6.4. Deregulated Expression of Human Genes by HPV Integration

Huebbers et al., investigated differences in human gene expression between oropharyngeal tumors with and without HPV integration (detected by APOT/DIPS PCR) [30]. They showed that AKR1C1 and AKR1C3 protein expression was upregulated in OPSCC with HPV integration. Upregulation of AKRs (compared to expression in the adjacent normal squamous epithelium) was also detected in HPV-negative OPSCC, most probably because of oxidative stress response, induced by mutations in the Keap1/Cul3/NRF2 system [30,86]. AKRs play a role in prostaglandin, steroid hormone, and retinoid metabolism. Furthermore, they are phase I detoxifying enzymes involved in the modification of chemotherapeutic drugs [76]. Interestingly, there are feedback loops between oxidative stress response and AKR1C expression with NRF2 binding to antioxidant response elements (ARE) in the promoter regions of the AKRCs increasing their expression [76]. Furthermore, the viral spliced isoform HPV16-E6*I was shown to interact with SP1-binding sites within the AKR1C1 promoter regions also resulting in increased AKR1C1 expression [86]. On the other hand, an increase in AKR1C1 and AKR1C3 protein expression results in decreased concentrations of retinoic acids, known inhibitors of NRF2 function, which subsequently also lead to NRF2 activation [87]. The activation of NRF2 consequently activates PI3K-AKT signaling, metabolic reprogramming, cell proliferation, insufficiency in autophagy, chemotherapy resistance as well as impaired DNA damage response [30,88,89]. It was also demonstrated by Huebbers et al. and Zhang et al. that HPV16-E6*I expression was upregulated significantly in OPSCCs with integrated viral genome [30,88]. Furthermore, in both of these studies, viral integration and E6*I overexpression are correlated with keratinocyte differentiation signatures. Similarly, Paget-Bailly et al. reported that ectopic expression of HPV16 E6*I induced deregulation of cellular genes participating in ROS metabolism, promoting viral integration by inducing genome instability [90]. The presence of E6 partially counteracts the impact of E6*I. Additionally, the above is also supported by studying a clinical cohort, where the subgroup of tumors overexpressing E6*I was associated with key cancer pathways linked to ROS metabolism [91]. However, further studies should be performed to understand how E6*I regulates genes associated with oxidative stress and how this impacts HPV-driven tumorigenesis [90].

Pannone et al., showed an association between HPV integration (detected by ISH) and Toll like receptor (TLR) 4 downregulation [92]. TLRs are predominantly involved in the innate immune response to pathogens including HPV and recognize Pathogen-associated Molecular Patterns (PAMPs) such as nucleic acids or proteins of viral origin, which serve as TLR activating ligands. [93]. Ligand bound TLR4 then triggers lipid raft flowing, resulting in a conformational change. This in turn leads to aggregation of NADPH oxidase subunits on these lipid rafts resulting in ROS production and increased HIF1α expression adding to the hypoxic tumor conditions [93]. TLR4 furthermore activates signaling cascades including tumor necrosis factor receptor-associated factor 3 (TRAF3) and nuclear factor kappa-light-chain-enhancer of activated B cells (NF-ϰB), which regulate the production of interferons (INF), inflammatory cytokines, and chemokines. However, in uterine cervical carcinomas and HPV-positive OPSCCs, a decrease in the TLR4 expression compared to normal epithelium is observed [92]. The viral proteins E6 and E7 have the property to interfere with innate immunity, e.g., by interacting with interferon regulator factor 3 (IRF-3) (E6) or IRF-1 (E7). As a result, HPV gains the ability to escape both innate and adaptive immune response and further avoid being recognized by Antigen Presenting Cells (APC)s [92].

The presence of episomal HPV DNA also showed to correlate with deregulation of pathways involved in immune response and cell survival in an indirect manner. Hajek et al. discovered that 85% of tumors with mutations in the genes *TRAF3* and *CYLD* (Cylindromatosis Lysine 63 Deubiquitinase) contained episomal HPV (data from The Cancer Genome Atlas) [78]. *TRAF3* is one of the most frequently mutated genes in HPV-positive HNSCCs (25% of HPV-positive tumors), but, remarkably, is not usually found to be mutated in their HPV-negative counterparts (2%) [94]. In addition, the tumor suppressor gene *CYLD* was found to be mutated in 11% of HPV-positive tumors. Both TRAF3 and CYLD play a role in both negatively regulating NF-ϰB canonical and noncanonical pathways while simultaneously stimulating a potent and first-line antiviral response through type I IFN signaling. Mutations in these genes will therefore lead to constitutive activation of NF-ϰB, which promotes cell survival and an impaired innate immunity against viral infections [68,95,96]. Moreover, it is suggested that maintenance of episomal HPV even pressures cells to mutate *TRAF3*/*CYLD*. These mutations might provide support for an alternative mechanism of HPV tumorigenesis in HNSCCs, not depending on viral integration into the host cell genome, to provoke a malignant transformation [78].

### 3.7. Subgroups of HPV-Positive Tumors Associated with Viral Integration Status

Recent studies have shown that HPV-positive tumors represent a heterogeneous group with respect to mRNA expression signatures as well as HPV integration status, with biological and clinical relevance. Two main subgroups have been characterized based on mRNA expression signatures, namely HPV-IMU and HPV-KRT (HPV-keratinocyte differentiation and oxidative reduction process) [2,88,97]. Molecular analyses revealed that the HPV-KRT subgroup more frequently contains integrated HPV (70–78% of the cases), shows a lower expression of E2/E4/E5, and has a higher ratio of spliced E6 compared to full length E6, which is in agreement with observations described above. Furthermore, this group was enriched for chromosome 3q amplifications and PIK3CA mutations. HPV-IMU tumors showed less integration (25–36% of the cases) and were enriched for chromosome 16q losses (detected by RNA sequencing).

Another study of Locati et al. identified three main clusters of HPV-positive tumors; Cl1 (immune-related), Cl2 (epithelial-mesenchymal transition-related), and Cl3 (proliferation-related) [98]. Tumors classified as Cl1 showed viral integration in 45% of the cases, whereas tumors classified as Cl2 and Cl3 showed 100% and 77% integration, respectively. In addition, the three clusters have been observed to have prognostic relevance, with Cl1 correlating to the best survival rate, and Cl2 to the worst survival rate. Knowledge on subtypes within HPV-positive tumors might contribute to patient selection for either de-escalation or personalized therapeutic approaches [11].

### 3.8. HPV Integration in Relation to Prognosis

The association of HPV integration with patient prognosis has been a topic of debate for several years [15]. More recent studies indicate an association of viral integration with unfavorable prognosis.

Nulton et al., demonstrated, using the expression of E2 as a marker for integration in TCGA HNSCC samples, that patients with fully episomal or a mixed form of HPV16 showed better survival than patients with integrated HPV16 as well as patients with HPV-negative HNSCCs [99]. Similarly, Hajek et al. observed that the HPV-positive subset of HNSCC in the TCGA database with mutations in the genes TRAF3 and CYLD were associated with the maintenance of episomal HPV and improved survival of patients [78]. For this association, they used the NGS determined integration data from the study of Parfenov et al. [60]. Moreover, Veitía et al. evaluated 80 fresh biopsies of head and neck cancer, mostly oral cavity, larynx, and oropharynx tumors, using E2/E6 qPCR. Of the 28 HPV16 positive samples, 86% displayed integration, possessed low viral load and correlated to poor prognosis. [100]. Supporting these results, Koneva et al. showed that patients with (RNAseq determined) integration-positive oropharyngeal and oral cavity tumors had statistically significant worse survival than patients with integration-negative tumors and similar survival as patients with HPV-negative HNSCCs [80]. Moreover, patients with integrated HPV were significantly older than patients with episomal HPV and comparable to HPV-negative patients, suggesting that older age was associated with worse survival [80,99].

In addition, Huebbers et al. showed that HPV integration in oropharyngeal tumors (analyzed with APOT- and DIPS PCR) was associated with upregulation of AKR1C1 and AKR1C3 expression [30]. Upregulation of AKR1C1 and AKR1C3 correlated with negative outcomes for both chemo- and radiotherapy in both overall and disease-free survival. Contrastingly, low expression of AKR1C1 and/or AKR1C3 was significantly correlated with favorable outcomes in surgical treatment. Intriguingly, viral integration also seems to be associated with a more progressive and persistent disease [101,102,103].

In contrast, both Vojtechova et al. and Lim et al. showed that there were no significant differences in survival between patients with episomal, mixed or integrated HPV16 in oropharyngeal tumors (*n* = 186 and *n* = 179, respectively) [104,105]. Vojtechova used three different detection techniques (E2 transcript breakpoint analysis, APOT, and Southern blotting). Lim et al. observed a trend towards better survival in patients with mixed HPV compared to patients with either episomal or integrated HPV; however, they used E2/E6 qPCR, possibly leading to overestimation of mixed viral physical status, as discussed before.

Recently, Pinatti et al., showed, using DIPS-PCR analysis on 35 tumors, mainly of the oropharynx, that HPV integration was correlated with favorable disease-specific survival when compared to patients without integration [106].

Overall, studies reporting on the correlation of viral integration with patient prognosis of HPV-positive HNSCCs have shown inconsistent results. As mentioned before, the technique used to detect viral integration is important to consider when interpreting the results of these studies. As an example, PCR for E2 and E6/7 expression might overestimate mixed physical status of HPV. Furthermore, studies often include tumors from different anatomical locations and relatively small patient groups.

## 4. Conclusions

In conclusion, a number of different technologies (including FISH, PCR, and NGS) have been used to determine the physical status of HPV in HNSCC, predominantly HPV16 in oropharyngeal tumors. Dependent on the viral detection strategy, HPV integration prevalence may differ. Results indicate that HPV integration is not simply a random event but rather prefers less protected and more accessible chromosomal regions, including highly transcribed (cancer) genes. Besides known mechanisms that can lead to DNA damage and subsequent viral integration, for example ROS, toxic agents, and inflammation, recent literature has provided evidence that APOBEC expression, induced by antiviral response, is doing so. Recent studies show that HPV integration affects both the viral and host genome, leading to constitutive expression of viral oncoproteins and deregulation of cellular (cancer) genes, possibly conferring additional neoplastic pressure. HPV integration appears to upregulate genes involved in metabolic pathways and immune evasion and downregulate genes involved in inflammation, apoptosis, and immune responses. On the other hand, episomal HPV was associated with mutations in *TRAF3* and *CYLD*. Although new data suggest a correlation between HPV integration and unfavorable prognosis, more genome-wide studies with a larger sample size, especially of oropharyngeal origin, are required. Ideally, a uniform detection method utilizing NGS technology should be applied, and integration results should be validated using multiple techniques, to further investigate the biological and clinical implications of HPV integration in HNSCC.

## Figures and Tables

**Table 1:** Advantages and disadvantages of techniques used to detect HPV integration.

Technique		Advantages	Disadvantages	Ref
In-situ hybridization (ISH)	(Fluorescence) in-situ hybridization ((F) ISH)	Highly sensitive Suitable for morphologically preserved isolated cells, histological tissue sections or chromosome preparations Relatively fast results within one day Relatively expensive with respect to PCR; relatively cheap with respect to sequencing Able to identify number of integration sites per nucleus Able to determine if integration site produces active transcripts (RNAse and DNAse pre-treatment)	Requires prior knowledge about sequence of interest, e.g., in case of human–virus colocalization Requires probe mixture to allow high-risk HPV detection, typing needs additional ISH experiment Cannot determine site of integration if only virus probe is used Cross-hybridization can occur when analyzing highly similar sequences (e.g., HPV6 and HPV11)	[26,27]
Polymerase Chain Reaction (PCR)	Quantitative or Real-Time PCR (qPCR, RT-PCR)	Highly specific Extremely sensitive Suitable for fresh frozen material Relatively cheap with respect to sequencing Able to detect viral load based on fluorescence timing	Less suitable for FFPE 1 material Cannot determine site of integration Cannot indicate physical status Cut-off for E2:E6/7-ratio is either less or strong discriminating Integration can occur in different genes: E2 is not always deleted, E1 can also be deleted	[28,29]
	Detection of Integrated Papillomavirus Sequences PCR (DIPS-PCR)	Suitable for fresh frozen material Relatively cheap with respect to sequencing Able to indicate physical status Able to determine site of integration.	Less suitable for FFPE material Aimed only at fractures in E2 Restriction enzyme is a limiting factor, since the site of integration into the human genome is unknown Digested fragment needs to be at correct length: too long fragments make it difficult to be accurately detected by PCR, too short fragments ensures that integration site remains unknown	[30,31,32,33]
	Amplification of Papillomavirus Oncogene Transcripts PCR (APOT-PCR)	Suitable for fresh frozen material Relatively cheap with respect to sequencing Able to indicate physical status Able to determine site of integration if integration occurred in a gene Able to determine if integration site produces active transcripts Highly accurate Highly sensitive, even with large number of samples Able to determine site of integration and viral copy number Able to identify both 5′ and 3′ end breakpoints through hybrid reads Little to no bias due to nature of technique	Less suitable for FFPE material Requires stable RNA of good quality Requires expression of active transcripts Cannot determine site of integration if integration occurred in an intergenic region or an intron due to alternative splicing	[30,31,32,33]
Next-Generation Sequencing (NGS)	RNASeq	Suitable for RNA from blood, fresh-frozen biopsy, FFPE, fine needle aspirates, core needle biopsies and single cells Able to deep profile the transcriptome Able to determine if integration site produces active transcripts Requires lower depth to find 3′ HPV breakpoints with respect to DNA-based NGS due to level of virus transcripts Unbiased approach to view entire RNA population	Cannot find 5′ ends of HPV breakpoints Cannot find HPV integrants that are transcriptionally repressed Can produce false 3′ calls with splice reads Depth may be reduced because of breadth of coverage	[21,26]
	Whole Genome Sequencing (WGS)	Suitable for genomic DNA (gDNA) from blood and fresh-frozen biopsy. Highly accurate Highly sensitive, even with large number of samples Able to determine site of integration and viral copy number Able to identify both 5′ and 3′ end breakpoints through hybrid reads Little to no bias due to nature of technique	Requires high read depth, deep sequencing and good coverage to find absolute integrant breakpoints Relatively expensive with respect to PCR and (F)ISH Relatively time consuming Cannot determine if HPV integrants are transcriptionally active	[21,34,35]
	Whole Exome Sequencing (WES)	Suitable for genomic DNA (gDNA) from blood, fresh-frozen biopsy Highly accurate Extremely sensitive, even with large number of samples Relatively cheap with respect to WGS due to limited target Able to obtain higher depth with respect to WGS due to limited target Able to determine site of integration and viral copy number Able to identify both 5′ and 3′ end breakpoints through hybrid reads Little to no bias due to nature of technique	Less suitable for FFPE material Requires high read depth, deep sequencing and good coverage to find absolute integrant breakpoints. Cannot identify integration sites in non-coding regions. Cannot determine if HPV integrants are transcriptionally active	[21,34,35]
	Capture-based assay	Suitable for genomic DNA (gDNA) and/or RNA from blood, fresh-frozen biopsy, DNA and RNA from FFPE, fine needle aspirates, and core needle biopsies. Able to determine site of integration and viral copy number Able to identify both 5′ and 3′ end breakpoints through hybrid reads Increases chance of finding HPV integration sites due to sequence capture Little to no bias due to nature of technique Can be adapted for additional methods, such as chromosome conformation studies	Requires high read depth, deep sequencing and good coverage to find absolute integrant breakpoints Requires individual probes for each HPV type Cannot determine if HPV integrants are transcriptionally active Excludes majority of host sequence	[21,36]
Emerging Techniques	Nanopore Sequencing	Imaging equipment is not required; hence the system can be scaled down to portable level On comparison to other massively parallel sequencers, the device is of much lower cost The captured DNA can be sequenced rapidly Long reads of DNA can be sequenced Able to sequence long repetitive DNA sequences and structural variants	Less suitable for FFPE ^1^ material Not suitable for single nucleotide variation detection Extremely high molecular weight DNA needed for library preparation The sequencer has the drawback of having high error rate ranging from 5% to 20%, based on the sort of molecules and methods of library preparation	[37]
	Targeted Locus Amplification	Suitable for purified gDNA from fresh-frozen tissues, fresh tissues and FFPE material Does not require detailed knowledge on locus sequence information Able to determine site of integration and viral copy number Able to identify both 5′ and 3′ end breakpoints through hybrid reads Increases chance of finding HPV integration sites due to sequence capture Relatively long reads of DNA can be sequenced (1 kb in FFPE up to 50–100 kb in fresh cells) surrounding a known/specific sequence/captured target enabling more robust analysis with respect to traditional/standard DNA-based NGS.	Requires high read depth, deep sequencing and good coverage to find absolute integrant breakpoints Complex and extensive integration profile may be challenging to map out completely. Integration sites could be missed in case of a large number of episomal HPV Requires individual probes for each HPV type Cannot determine if HPV integrants are transcriptionally active Excludes majority of host sequence	[38]

^1^ FFPE = formalin fixed paraffin embedded.

## Data Availability

No new data were created or analyzed in this study. Data sharing is not applicable to this article.

## Appendix A. Search Terms Used for Systematic PubMed Search

- ((Head[Tiab] OR neck[Tiab] OR “head and neck” [Tiab] OR “head-neck” OR “head-and-neck” [Tiab] OR oral[Tiab] OR pharyn*[Tiab] OR OR laryn*[Tiab] OR oropharyn*[Tiab] OR nasopharyn*[Tiab] OR hypopharyn*[Tiab] OR throat[Tiab] OR glotti*[Tiab] OR mouth[Tiab] OR palate[Tiab] OR gingiva*[Tiab] OR lip[Tiab] OR cheek[Tiab] OR bucc*[Tiab] OR gum*[Tiab] OR tonsil*[Tiab] OR tongue[Tiab] OR nasal[Tiab] OR paranasal[Tiab] OR sinus[Tiab] OR saliv*[Tiab] OR ent[Tiab] OR aerodigestive[Tiab] OR “aero digestive” [Tiab] OR aero-digestive[Tiab])
- AND (cancer* OR carcinoma* OR neoplas* OR tumor* OR tumour* OR malignan* OR SCC OR “Neoplasms”[Mesh])) OR (hnscc[Tiab] OR scchn[Tiab] OR “Head and Neck Neoplasms”[Mesh])
- AND
- (“Human papilloma virus” [Tiab] OR “Human papilloma viruses” [Tiab] OR “Papillomavirus, Human” [Tiab] OR “Human papillomavirus” [Tiab] OR HPV [Tiab] OR HR-HPV [Tiab] OR “High-risk HPV” [Tiab] OR “HPV infection*” [Tiab] OR “Papillomavirus Infections/pathology” [Mesh])
- AND
- (integration [Tiab] OR “virus integration*” [Tiab] OR “virus integration” [Mesh] OR “Viral integration*” [Tiab] OR “human papillomavirus integration” [Tiab] OR “HPV integration” [Tiab] OR “genome integration” [Tiab] OR “viral DNA integration” [Tiab] OR “virus DNA integration” [Tiab] OR “HPV DNA integration” [Tiab] OR “HPV insertion*” [Tiab] OR “Human papillomavirus insertion*” [Tiab])
